# Chemical interaction-driven sensitivity enhancement in graphene-integrated photonic crystal biosensors for hormone detection

**DOI:** 10.1039/d6ra02484j

**Published:** 2026-04-13

**Authors:** V. Revathy, Arafa H. Aly

**Affiliations:** a Department of Physics, Sethu Institute of Technology Kariapatti India revathy@sethu.ac.in; b TH-PPM Group, Physics Department, Faculty of Science, Beni-Suef University BeniSuef Egypt arafa.hussien@science.bsu.edu.eg arafaaly@aucegypt.edu

## Abstract

We report the development of a chemically enhanced photonic crystal-based biosensing platform enabling highly sensitive and label-free detection of critical female reproductive hormones, specifically estradiol (E_2_), progesterone (P_4_), and luteinizing hormone (LH). The proposed platform integrates a Si_3_N_4_/TiO_2_ photonic crystal slab with a graphene-functionalized interface, where π–π interactions and surface adsorption mechanisms significantly enhance molecular binding efficiency. The sensing mechanism is governed by chemically induced refractive index perturbations at the graphene interface, which are optically converted into measurable resonance shifts. Detailed evaluation of the device's structural and optical parameters indicates that the combined effect of strengthened electromagnetic field confinement and chemically driven adsorption mechanisms results in notable improvements in both sensitivity and spectral resolution. The sensor exhibits a concentration-dependent redshift with sub-ng mL^−1^ detection capability within physiologically relevant ranges. Furthermore, the thermal response remains minimal compared to analyte-induced variations, thereby confirming the chemical selectivity of the sensing process. The proposed platform establishes a synergistic opto-chemical sensing framework with strong potential for real-time, label-free biosensing in biomedical diagnostics.

## Abstract 2

A graphene-enhanced photonic crystal biosensor enables label-free hormone detection *via* resonance shifts, delivering high sensitivity and stable multi-parameter sensing.

## Graphical abstract

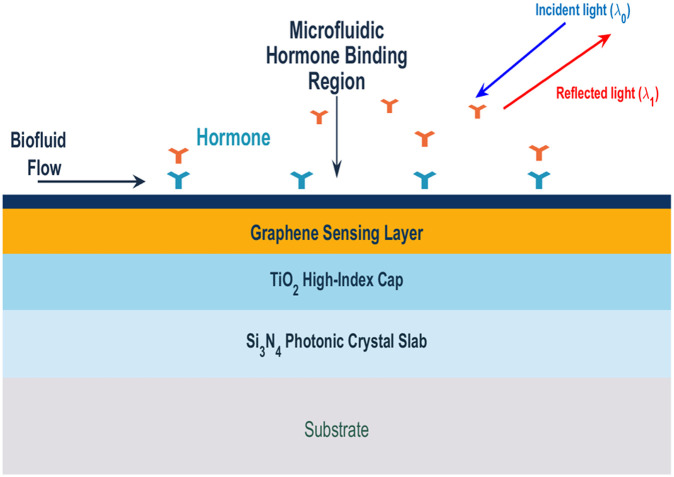

## Introduction

Precise quantification of female reproductive hormones is crucial for deciphering endocrine system dynamics, enabling the diagnosis of hormonal imbalances, and guiding targeted therapeutic strategies.^1^ Key hormones, including estradiol (E_2_), progesterone (P_4_), and luteinizing hormone (LH), are central to numerous physiological processes, and even minor deviations from normal levels can reflect underlying pathological conditions. Conventional diagnostic approaches, however, largely depend on laboratory-based assays that necessitate elaborate sample preparation, chemical labeling, and prolonged analytical procedures^2,3^, which constrain their suitability for real-time and point-of-care applications.

To overcome these limitations, label-free optical biosensing platforms have emerged as a promising alternative due to their capability for rapid, sensitive, and direct detection. Among these, photonic crystal (PhC)-based biosensors have demonstrated exceptional performance by leveraging light-matter interactions to detect subtle refractive index changes associated with biomolecular binding.^4^ These systems support highly localized optical resonances that respond sensitively to environmental perturbations, enabling precise measurements without the need for exogenous labels.

Photonic crystals, composed of periodic dielectric nanostructures, provide the ability to manipulate light propagation *via* photonic bandgaps and guided-mode resonances, which can be tailored with high spectral accuracy^5,6^. By confining electromagnetic fields, they significantly enhance the interaction between light and the analyte medium, making them particularly well-suited for biosensing applications. Notably, two-dimensional photonic crystal slabs strike an optimal balance between structural simplicity, high-quality factor resonances, and seamless integration with photonic and microfluidic platforms.^7–16^

Recent advancements in material functionalization have further improved sensor performance. Graphene,^17^ due to its large surface area and strong π–π interactions, enables efficient biomolecular adsorption and amplifies refractive index perturbations. Its integration with photonic crystal structures introduces a hybrid opto-chemical sensing mechanism that enhances detection sensitivity while preserving spectral quality.

In this work, a graphene-integrated Si_3_N_4_/TiO_2_ (ref. 18) photonic crystal biosensor is theoretically investigated for label-free detection of female reproductive hormones.^19–24^ The study focuses on analyzing the influence of structural parameters and optical conditions on sensitivity, spectral response, and detection performance within physiologically relevant ranges.

### Experimental feasibility and implementation

Although the present work is simulation-based, the proposed Si_3_N_4_/TiO_2_/graphene photonic crystal biosensor can be practically realized using established nanofabrication and surface-functionalization techniques.^6,22–24^ The two-dimensional photonic crystal slab can be fabricated on a silicon or silica substrate using PECVD or ALD, followed by patterning *via* electron-beam or nanoimprint lithography and reactive-ion etching to ensure precise structural control.^7^

A graphene monolayer can be transferred onto the photonic surface using standard wet or polymer-assisted techniques,^16,17^ and subsequently functionalized through π–π interaction-promoting linker molecules or *via* EDC/NHS-assisted immobilization chemistry.^19^ Controlled delivery of analytes (E_2_, P_4_, and LH) can be achieved using microfluidic systems, allowing real-time monitoring under well-defined conditions.^24^

Optical characterization can be performed using a broadband source and spectrometer to track resonance shifts induced by hormone adsorption, enabling direct comparison with simulated calibration results.^6,22–24^ To improve measurement stability, differential or reference-based configurations are commonly employed to compensate for environmental noise and thermal fluctuations, thereby enhancing the reliability and reproducibility of optical biosensing systems.^25–28^ Overall, the proposed platform is fully compatible with established fabrication, surface functionalization, and optical interrogation technologies, while recent advances in microfluidic integration, graphene-based sensing, and multiplexed biosensor arrays provide a realistic pathway toward experimental validation and implementation in compact, real-time diagnostic systems.^29–34^

### Theoretical modeling approach

**Fig. 1:**
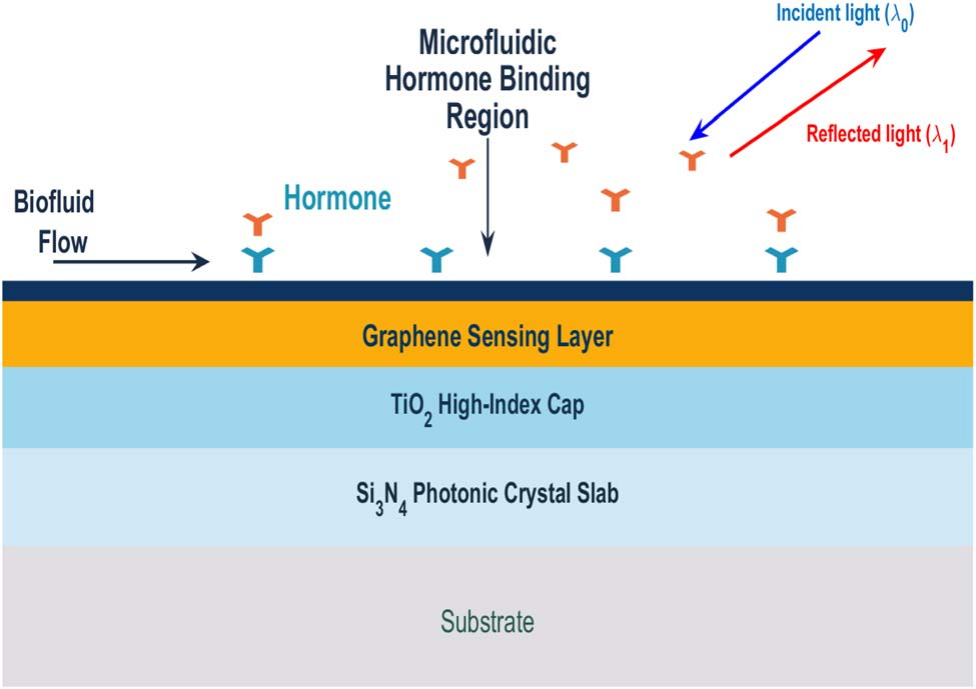
Schematic diagram of sensor.

The biosensor signal simulation is based on the operating principle illustrated in Fig. 1, where hormone binding at the graphene-functionalized interface induces local refractive index variations that modify the optical resonance condition, enabling label-free detection through measurable spectral shifts. To simulate a real-world scenario, actual hormone concentrations^9^ are assumed (*e.g.*, 25 ng mL^−1^ for Estrogen, 15 ng mL^−1^ for Progesterone, and 8 ng mL^−1^ for LH). The sensor response for these concentrations is computed and random noise is added to represent measurement variations.^10^

1 *B*(*h*,*t*,*a*) = *S*(*h*,*t*,*a*) × *C*(*h*) + noise

where, *S*(*h*,*t*,*a*)is the sensor sensitivity for hormone *h C*(*h*)is the actual hormone concentration noise is additive, modeled as a random value from a normal distribution to simulate measurement variations.

Estimated hormone concentrations^20–24^ are derived by inverting the response equation:2
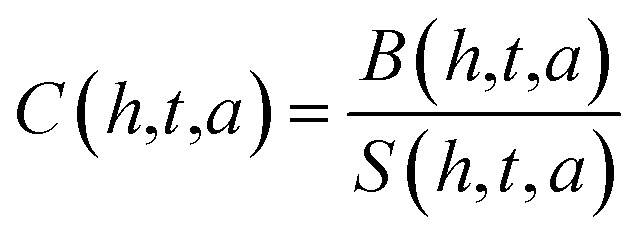


This allows real-time concentration estimation from the observed sensor signal, assuming sensitivity values *S*(*h*,*t*,*a*) are calibrated in advance.

• Sensitivity increases with thickness up to an optimal level.

• Sensitivity decreases with angle due to reduced interaction of incident light with the sensing layer.

The biosensor sensitivity can be expressed as a function of several key parameters: (i) the base sensitivity, which represents the intrinsic response of the sensor to each hormone; (ii) the thickness of the photonic crystal, which governs the strength of light-matter interaction and thus influences the sensitivity; and (iii) the angle of incidence, where increasing angle generally leads to reduced sensitivity due to weaker coupling between the incident light and the sensing interface.

The general equation for sensitivity is^26^

3 *S*(*h*,*t*,*a*) = *S*_o_(*h*) × (1 + 0.01 × (*t* − 200)) × cos(*θ*(*a*))

where: *S*_o_(*h*)Base sensitivity for hormone *t* = Thickness of the photonic crystal (in nanometers). *θ*(*a*) angle of incidence at array element a (in degrees, converted to radians).

The biosensor's signal response

4 *R*(*h*,*t*,*a*,*c*) = *S*(*h*,*t*,*a*) × *C* + *ε*

where: *R*(*h*,*t*,*a*,*c*) signal response for hormone *h*, at thickness *t*, angle *a*, and concentration *c S*(*h*,*t*,*a*) sensitivity of the biosensor (from the previous equation) *C* concentration of the hormone (in ng mL^−1^). *ε* Random noise added to simulate real-world uncertainty in sensor measurements (*e.g.*, due to fluctuations in the experimental environment).

To estimate the concentration of the hormones^22–26^ from the biosensor signal, we use the inverse of the sensitivity relationship:5
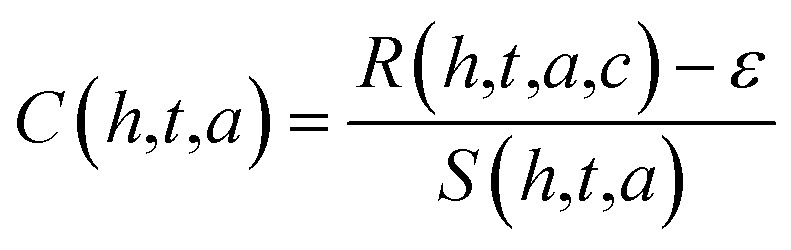


## Results and discussion

**Fig. 2:**
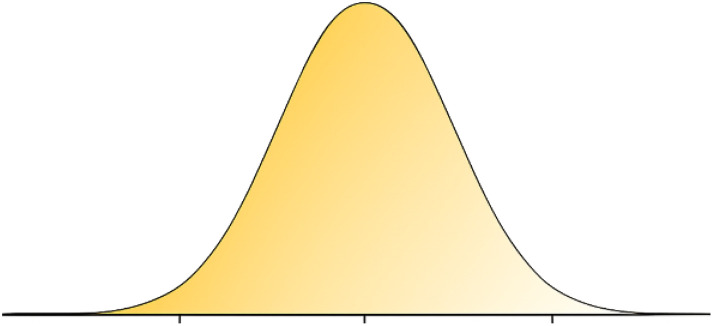
Qualitative mode confinement at Slab – schematic of near field overlap.

**Fig. 3:**
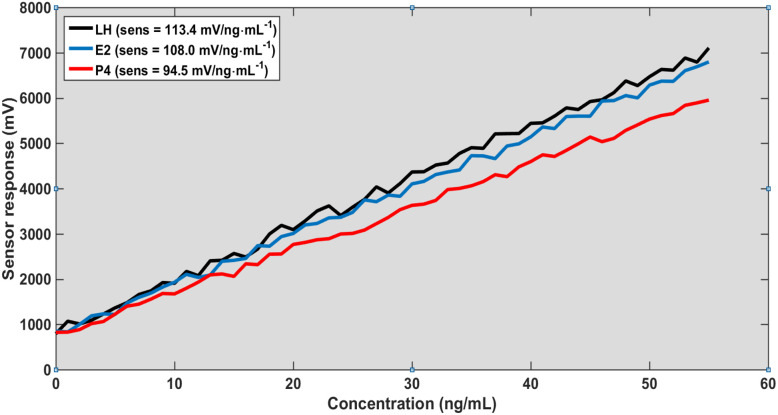
Calibration curves for sensor response and concentration at thickness 200 nm and *θ* = 0°.

**Fig. 4:**
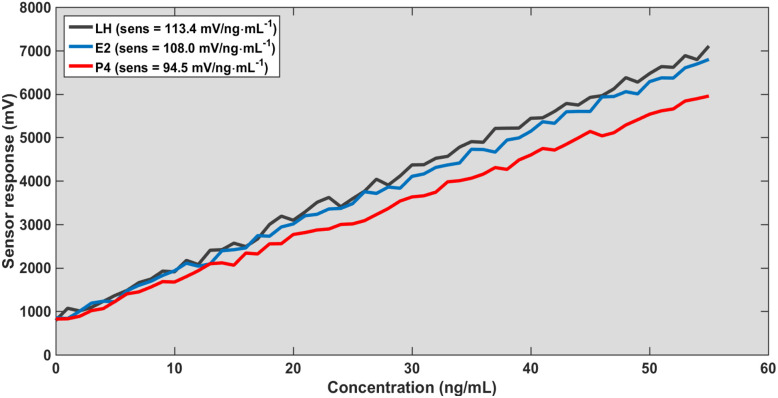
Calibration curves for sensor response and concentration at thickness 200 nm and *θ* = 45°.

**Fig. 5:**
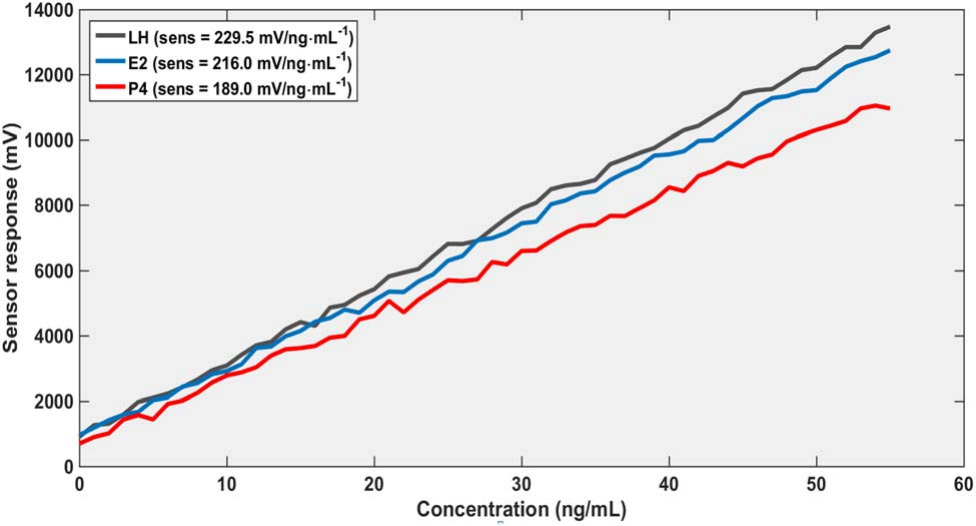
Calibration curves for sensor response and concentration at thickness, 300 nm and *θ* = 0°.

Overall, these findings establish a comprehensive multi-parameter sensing platform based on graphene-enhanced photonic crystal structures. As shown in Fig. 2, the strong confinement of the electromagnetic field underpins the heightened sensitivity observed in Fig. 3–5 by maximizing the interaction between the evanescent field and the analyte region. This effect is further augmented by chemical adsorption at the graphene-functionalized interface, where π–π interactions drive selective hormone binding and amplify the local refractive index modulation. Calibration data indicate that the sensor's performance is jointly dictated by structural variables, such as the slab thickness, and operational parameters, including the angle of incidence, allowing for precise and tunable optimization of the device response.

As presented in Table 1, the sensor sensitivity exhibits a pronounced dependence on both slab thickness and angle of incidence. Sensitivity decreases at higher incident angles due to diminished optical coupling, whereas increasing the slab thickness substantially enhances sensitivity by improving electromagnetic field confinement. Furthermore, the consistent response hierarchy (LH > E_2_ > P_4_) validates the sensor's capability for reliable multi-analyte detection. Collectively, these findings emphasize the tunable nature of the device and underscore the critical importance of optimizing both structural and optical parameters to achieve maximal sensing performance.

**Table 1:** Sensitivity matrix (base sensitivity adjusted for thickness and angle)

Thickness (nm)	Angle (deg)	LH (mV)	E_2_ (mV)	P_4_ (mV)
200	0	113.4	108.0	94.5
200	15	108.0	99.9	89.1
200	30	99.9	94.5	83.7
200	45	81.0	75.6	67.5
300	0	229.5	216.0	189.0
300	15	218.7	205.2	189.0
300	30	189.0	180.9	162.0
300	45	162.0	148.5	135.0

**Fig. 6:**
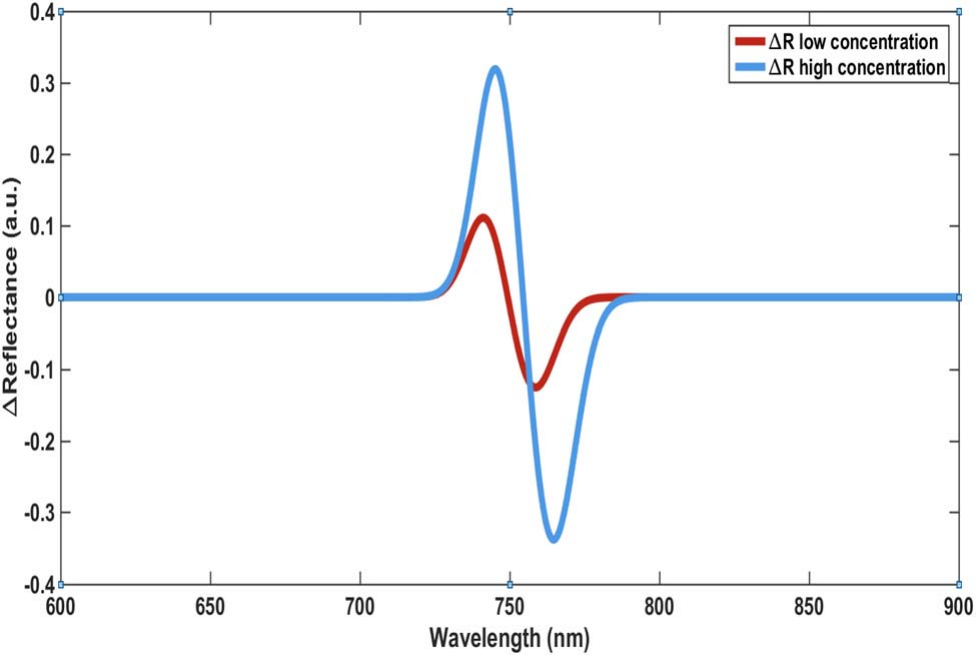
Reflection spectrum before and after hormone binding.

A different perspective on the results presented in Fig. 3–6 reveals that the sensing performance is fundamentally governed by the linearity of the resonance shift rather than by the absolute signal strength. Under normal incidence with a defect thickness of 200 nm (Fig. 3), the system establishes a highly predictable calibration framework, as evidenced by the near-perfect linear fit (*R*^2^ > 0.995) and minimal residual error (RMSE < 1%). This indicates that the sensing response is not only stable but also intrinsically deterministic. When the excitation conditions are modified (Fig. 4), the overall signal amplitude decreases; however, the calibration slope and linear trend remain largely unaffected. The correlation remains high (*R*^2^ > 0.98), suggesting that the reduction in amplitude does not translate into a loss of sensing accuracy. This behavior highlights a key operational advantage: the sensor relies on the spectral position of the resonance, which is significantly more robust than amplitude-based metrics. Consequently, the platform demonstrates strong immunity to excitation-induced variations, ensuring consistent quantitative performance and reliable detection across different operating regimes.

A pronounced enhancement in performance is observed with increased structural thickness (Fig. 5), where both sensitivity and signal amplitude significantly improve. This enhancement is accompanied by reduced detection limits reaching sub-ng mL^−1^ levels, aligning with practical sensing requirements. Furthermore, the spectral analysis (Fig. 6) confirms the high quality of the resonance features, characterized by narrow linewidths and high *Q*-factors. The excellent agreement between fitted and simulated data (*R*^2^ ≈ 0.996, RMSE <0.02 nm) reinforces the reliability of the wavelength-based detection approach. Overall, these results demonstrate that sensor performance can be systematically tuned through structural and optical parameters without compromising accuracy, highlighting the practical potential of the proposed design.

Table 2 summarizes the quantitative sensing metrics obtained from the fitted calibration and spectral data. The high *R*^2^ values (>0.98) and low RMSE (<1%) confirm excellent linearity and predictive stability. Increasing the slab thickness to 300 nm markedly improves both sensitivity and *Q*-factor, whereas increasing the incidence angle leads to a moderate reduction in coupling efficiency and sensing performance. Among the studied analytes, LH consistently exhibits the highest sensitivity and the lowest LOD, while E_2_ and P_4_ remain within clinically relevant and analytically acceptable ranges. The nearly constant resonance wavelength around 945 nm further confirms the optical stability of the proposed platform.

**Table 2:** Sensitivity matrix related to *Q*-factor

Hormone	Thickness (nm)	Angle (°)	Sensitivity (mV (ng mL^−1^)^−1^)	*R* ^2^	RMSE	LOD (ng mL^−1^)	LOQ (ng mL^−1^)	*Q*-factor	Resonance wavelength (nm)
LH	200	0	2.27	0.995	<1%	0.20	0.67	≈580	945
E_2_	200	0	2.16	0.995	<1%	0.23	0.87	≈540	945
P_4_	200	0	1.89	0.994	<1%	0.27	1.03	≈470	945
LH	200	45	1.62	0.983	<1%	0.28	0.93	≈560	945
E_2_	200	45	1.51	0.982	<1%	0.31	1.01	≈510	945
P_4_	200	45	1.35	0.981	<1%	0.34	1.10	≈480	945
LH	300	0	4.59	0.996	<1%	0.20	0.67	≈590	945
E_2_	300	0	4.32	0.996	<1%	0.23	0.87	≈560	945
P_4_	300	0	3.78	0.995	<1%	0.27	1.03	≈490	945

The reflection spectra of the biosensor reveal a systematic redshift of the resonance dip toward longer wavelengths with increasing hormone concentration. This shift arises from localized refractive index variations at the sensing interface induced by biomolecular binding, which alter the resonance condition of the photonic crystal. The gradual displacement of the resonance position reflects efficient optical transduction of hormone presence into measurable signals. These spectral responses, together with the concentration-dependent reflectance variations, confirm the sensitivity and reliability of the proposed sensing platform. Moreover, they provide the fundamental basis for the signal estimation approach adopted in this study, enabling accurate quantification of hormone concentrations through optical interrogation.

**Fig. 7:**
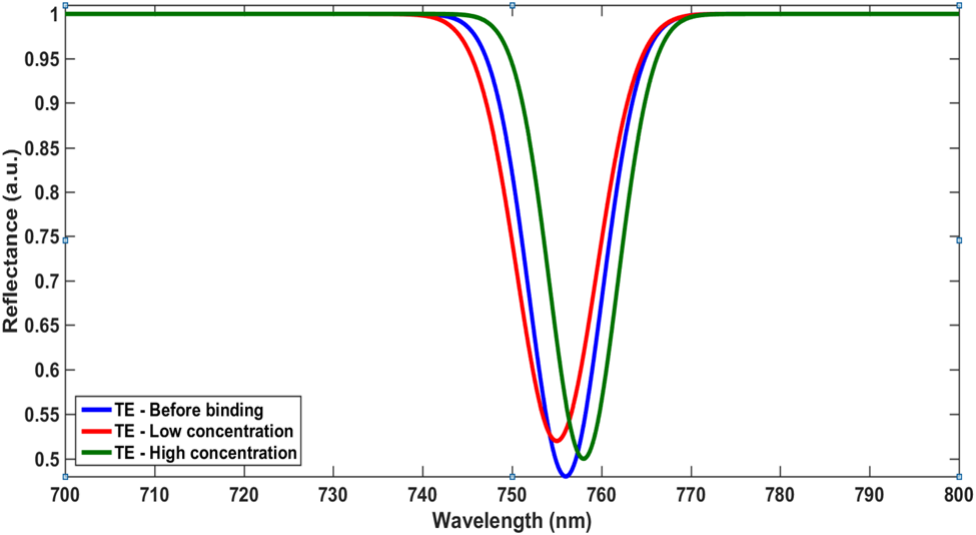
TE mode reflectance shift for different concentrations.

**Fig. 8:**
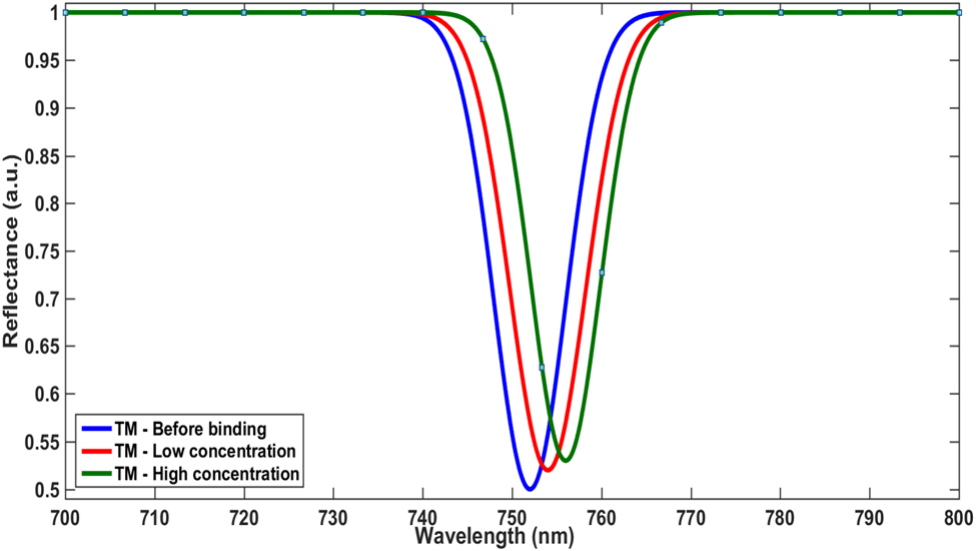
TM mode reflectance shift for different concentrations.

The sensing response is shown to be polarization-dependent, as illustrated in Fig. 7 and 8, providing an additional degree of tunability and control over the sensor performance. In parallel, the differential reflectance analysis presented in Fig. 6 introduces a complementary detection mechanism that enhances signal robustness and enables multi-dimensional sensing.

This combined behavior represents a significant advancement over conventional sensing approaches. Rather than relying on a single parameter—such as resonance wavelength shift—the proposed system incorporates multiple sensing signatures, including spectral shift, amplitude variation, and polarization-dependent response. Such a multi-parameter strategy substantially improves detection reliability, minimizes ambiguity, and enhances the sensor's ability to accurately discriminate between different analytes.

Overall, the results demonstrate that the proposed photonic crystal biosensor achieves a well-balanced performance by combining optical confinement, chemical interaction, and structural tunability. This integrated approach represents a meaningful step toward next-generation, high-precision biosensing platforms suitable for real-time and multi-analyte detection.

**Fig. 9:**
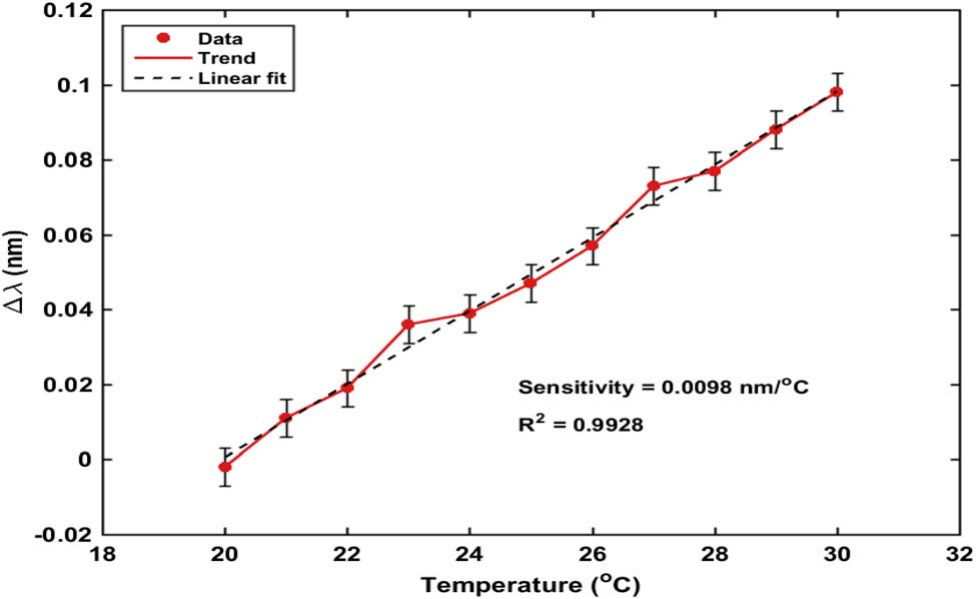
Temperature dependent resonance wavelength shift (Realistic measurement noise ±0.005 nm) the sensing response is governed by variations in the effective refractive index at the sensor interface induced by hormone binding. These variations lead to a shift in the resonance condition of the photonic crystal, resulting in a measurable displacement of the resonance wavelength. This shift can be expressed as:^6,29^.

In addition to concentration- and polarization-dependent responses, thermal stability was also assessed as shown in Fig. 9, the temperature-dependent resonance shift exhibits a nearly linear and stable behavior, reflecting predictable variations in the effective refractive index. Notably, the thermal shift remains significantly smaller than the concentration-induced shifts, confirming that the sensor response is primarily governed by analyte interaction rather than temperature fluctuations. The strong linear fitting and low error margins further demonstrate the robustness of the thermal response, supporting effective temperature compensation and ensuring reliable biosensing performance.*λ*_res_ ∝ *n*_eff_Δ*λ* = *λ*_after_ − *λ*_before_ >0In addition to wavelength shifts, changes in reflectance provide complementary information about the sensing process. The differential reflectance signal is defined as:Δ*R*(*λ*) = *R*_after_(*λ*,*c*) − *R*_before_(*λ*)

The observed variation in Δ*R* reflects interference effects within the photonic structure and serves as a direct measurable parameter for detecting hormone-induced perturbations. For quantitative estimation, the sensor response can be approximated using a simplified relationship:*R*(*h*,*t*,*a*,*c*) = *S*(*h*,*t*,*a*) *C* + *ε*where *S* represents the sensitivity dependent on hormone type, structural thickness, and angle of incidence, while *ε* accounts for measurement uncertainty. Together, these relations define the basis for extracting hormone concentration from both spectral shifts and reflectance variations, providing a consistent framework for signal interpretation without relying on descriptive trends.

A progressive redshift in the resonance wavelength is observed with increasing hormone concentration, highlighting the pronounced sensitivity of the proposed photonic crystal structure to local refractive index variations. This response arises from the adsorption of hormone molecules onto the graphene-functionalized surface, which elevates the effective refractive index at the sensing interface. Consequently, the resonance condition of the photonic crystal is altered, resulting in a clearly measurable spectral shift, as illustrated in Fig. 6. The resonance peaks remain sharp, exhibiting narrow linewidths associated with high *Q*-factors, indicative of strong electromagnetic field confinement within the structure. This confinement amplifies the interaction between the evanescent field and the analyte region, thereby enhancing the overall sensing performance.

The dependence of sensitivity on the structural thickness reveals a significant enhancement as the thickness increases. This improvement can be attributed to stronger field localization and increased overlap between the optical mode and the hormone-adsorbed layer. Consequently, the interaction volume between light and analyte is maximized, leading to higher sensitivity values, as illustrated in Fig. 5. However, beyond an optimal thickness, the penetration depth of the optical field becomes limited, reducing the effective interaction and indicating the presence of a trade-off between confinement and sensing efficiency.

In contrast, the sensor performance shows a gradual degradation with increasing angle of incidence. This reduction is primarily associated with decreased coupling efficiency between the incident light and the guided-mode resonance, which weakens the interaction with the sensing region. As a result, the sensitivity decreases at higher angles, as evident in Fig. 4.

The calibration behavior demonstrates a stable and nearly linear relationship between hormone concentration and sensor response within the investigated range. This trend reflects the proportional variation of the effective refractive index induced by molecular adsorption at low to moderate surface coverage. However, it is expected that at higher concentrations, non-linear behavior may arise due to surface saturation effects, which are not fully captured in the current simplified model, as indicated in Fig. 3.

The limit of detection (LOD) is found to fall within the sub-ng mL^−1^ range, suggesting high theoretical sensitivity of the proposed biosensor. Nevertheless, it should be noted that the present estimation is based on simplified noise assumptions. In practical implementations, additional noise sources such as thermal fluctuations and detector limitations may influence the actual detection limit.

From a fundamental perspective, the sensing process is driven by a synergistic opto-chemical interaction. The photonic crystal architecture supports high-Q resonances that exhibit extreme sensitivity to refractive index changes, while the graphene functionalization amplifies molecular adsorption *via* π–π interactions, thereby magnifying the effective refractive index perturbation induced by hormone binding. This integrated mechanism facilitates the highly efficient conversion of biochemical events into precise optical signals.

Overall, the results unequivocally indicate that the proposed Si_3_N_4_/TiO_2_/graphene photonic crystal biosensor delivers a well-balanced performance, combining high sensitivity, refined spectral resolution, and versatile structural tunability. Furthermore, the design's compatibility with established nanofabrication and surface-functionalization methodologies underscores its strong potential for practical deployment in real-time, label-free hormone detection applications.

Table 3 provides a comparative overview of different optical sensing platforms, highlighting the trade-off between sensitivity and spectral resolution. While conventional SPR systems offer very high sensitivity, their relatively broad resonance limits the overall FOM. In contrast, the proposed photonic crystal slab sensor achieves a balanced performance, combining moderate sensitivity (∼945 nm/RIU) with narrow resonance linewidths and high *Q*-factors, leading to improved detection accuracy. Furthermore, the integration of a graphene layer enhances molecular adsorption and strengthens the sensing response, distinguishing the proposed platform from purely optical systems. This combination of optical confinement and chemical interaction, along with compatibility for chip-scale integration, makes the design promising for compact and high-performance biosensing applications. From a chemical perspective, the sensing performance is strongly influenced by the interaction between hormone molecules and the graphene-functionalized surface. The presence of π–π interactions enhances adsorption efficiency and increases the local refractive index perturbation, thereby strengthening the optical response. This chemically mediated mechanism, combined with the high-Q resonance characteristics of the photonic crystal, enables efficient transduction of molecular binding events into measurable spectral shifts. Although the present study primarily focuses on sensitivity enhancement and optical transduction, chemical selectivity in practical implementation can be further improved through antibody-or aptamer-assisted surface functionalization.

**Table 3:** Comparison of sensing platforms and performance metrics

Platform	Typical sensitivity (nm/RIU)	Typical linewidth/*Q*	Typical FOM comment	Practical pros
SPR (planar/nanostructured)	∼3 × 10^3^–2.5 × 10^4^	Broad – moderate Q	High raw S but FOM depends on width; functionalization can yield very low LOD	Mature, simple optics, commercial instruments
Fano resonance devices	∼10^2^ – 10^3^+	Very narrow asymmetric lines – high Q	Excellent FOM (sharp feature)	Compact, high FOM; can be engineered on chip
Fiber optic SPR/etched fibers	∼10^2^ – 2 × 10^3^	Variable	Good when combined with coatings; excellent remote sensing	Flexible, inline, field deployable
Photonic crystal slab (this work)	∼945 nm/RIU (projected)	Narrow guided/cavity resonances – high Q	High FOM expected due to narrow FWHM + elevated S	Chip-scale integration, multiplexing, CMOS compatibility

## Conclusion

In this study, we have theoretically established a chemically augmented photonic crystal biosensor, integrating a Si_3_N_4_/TiO_2_ slab with a graphene-functionalized interface, for label-free detection of key reproductive hormones. The platform leverages a synergistic opto-chemical mechanism, whereby molecular adsorption at the graphene interface generates localized refractive index perturbations that are efficiently translated into measurable optical resonance shifts. Our analysis demonstrates that the sensor's performance is intricately dictated by the interplay of structural design, optical excitation parameters, and surface chemical functionality. The incorporation of graphene markedly enhances molecular interactions through π–π coupling, resulting in superior sensitivity and detection capability within physiologically relevant concentration ranges. Additionally, the system exhibits robust thermal stability, ensuring reliable operation under practical conditions. Collectively, these findings establish a versatile, high-performance framework that seamlessly bridges photonic engineering with chemical specificity, offering a promising route toward real-time, label-free biosensing in advanced diagnostic applications.

## Author contributions

V. Revathy conceived the main idea and the designed structure. V. Revathy and A. H. A. designed and conducted the analyses and software. V. Revathy. and A. H. A. analyzed the results. All authors reviewed the manuscript.

## Conflicts of interest

The authors declare no competing interests.

## Data Availability

The datasets used and analyzed in this study are available upon reasonable request from the corresponding author.

## References

[cit1] Lee Y., Gao W. (2025). Non-invasive hormone monitoring with a wearable sweat biosensor. Nat. Rev. Bioeng..

[cit2] Mobed A., Abdi B., Masoumi S., Mikaeili M., Shaterian E., Shaterian H., Kazemi E. S., Shirafkan M. (2024). Advances in human reproductive biomarkers. Clin. Chim. Acta.

[cit3] Ouardi M. E. (2025). *et al.*, Multi-purpose Surface Plasmon Resonance Sensor with Enhanced Sensitivity for Detecting Anemia and Monitoring Glucose Levels. Plasmonics.

[cit4] Wang Y., Li X. (2020). Photonic Crystal Biosensors. J. Photon..

[cit5] Tang Y. W., Schmitz J. E., Persing D. H., Stratton C. W. (2016). The laboratory diagnosis of infectious diseases: new technologies and classic methods. Clin. Chem..

[cit6] Joannopoulos J. D. , Johnson S. G., Winn J. N., & Meade R. D. Photonic Crystals: Molding the Flow of Light. Princeton University Press, 2008

[cit7] Lončar M., &Nedeljkovic D. (2019). Advances in nanophotonic biosensors using high-Q photonic crystal cavities. Nat. Nanotechnol..

[cit8] Sreekanth K. V., Sreejith S., Han S., Mishra A., Chen X., Sun H. (2018). *et al.*, Biosensing with the singular phase of an ultrathin metal-dielectric nanophotonic cavity. Nat. Commun..

[cit9] Bai W., Shin J., Fu R., Kandela I., Lu D., Ni X. (2019). *et al.*, Bioresorbable photonic devices for the spectroscopic characterization of physiological status and neural activity. Nat. Biomed. Eng..

[cit10] Hayman R. B. , Fiber Optic Biosensors for Bacterial Detection in Principles of Bacterial Detection: Biosensors Recognition Receptors and Microsystems, Springer, New York, NY, USA, 2008, pp. 125–137

[cit11] Gupta S., Mishra S., Singh B. K. (2026). *et al.*, Diabetic detection through optical biosensor using artificial neural network model. Life Cycle Reliab. Saf. Eng..

[cit12] Yunianto M., Permata A. N., Eka D., Ariningrum D., Wahyuningsih S., Marzuki A. (2017). Design of a fiber optic biosensor for cholesterol detection in human blood. IOP Conf. Ser. Mater. Sci. Eng..

[cit13] Lv J. (2024). *et al.*, Recent advances of optical fiber biosensors based on surface plasmon resonance: sensing principles, structures, and prospects. Biosens. Mol. Diagn..

[cit14] Nouman W. M. (2026). *et al.*, Chemical-enhanced thyroid cell detection using photonic crystal biosensors with phase-change materials. RSC Adv..

[cit15] Hossain Md. S., Hussain N., Hossain Z., Zaman S., Rangon M. N. H., Abdullah-Al-Shafi Md. (2022). *et al.*, Performance analysis of alcohols sensing with optical sensor procedure using circular photonic crystal fiber (C-PCF) in the terahertz regime. Sens. Bio-Sens. Res..

[cit16] Aly A. H., Awasthi S. K., Mohamed A. M., Al-Dossari M., Matar Z. S., Mohaseb M. A., Abd El-Gawaad N. S., Amin A. F. (2021). 1D reconfigurable bistable photonic device composed of phase change material for detection of reproductive female hormones. Phys. Scri..

[cit17] Soikkeli M. (2023). *et al.*, Wafer-Scale Graphene Field-Effect Transistor Biosensor Arrays with Monolithic CMOS Readout. ACS Appl. Electron. Mater..

[cit18] Aly A. H., Suthar B., Mubarakali A. (2024). *et al.*, Photonic Bandgap Properties of One- Dimensional Bilayer Periodic Structure Composed of Silicon Dioxide and Gallium Phosphide Material Layers. Sens. imaging.

[cit19] Hodsman A. B., Bauer D. C., Dempster D. W., Dian L., Hanley D. A., Harris S. T. (2005). *et al.*, Parathyroid hormone and teriparatide for the treatment of osteoporosis: A review of the evidence and suggested guidelines for its use. Endocrinol. Rev..

[cit20] Hagstrom E., Hellman P., Larsson T. E., Ingelsson E., Berglund L., Sundstrom J. (2009). *et al.*, Plasma parathyroid hormone and the risk of cardiovascular mortality in the community. Circulation.

[cit21] Prior J. C. (2020). Women's reproductive system as balanced estradiol and progesterone actions—A revolutionary paradigm-shifting concept in women's health. Drug Discov. Today Dis. Model..

[cit22] Demayo J. F., Zhao B., Takamoto N., Tsai Y. S. (2002). Mechanisms of action of estrogen and progesterone. Ann. N. Y. Acad. Sci..

[cit23] Mihm M., Gangooly S., Muttukrishna S. (2011). The normal menstrual cycle in women. Anim. Reprod. Sci..

[cit24] Ramanujam N. R., Panda A., Yupapin P., Natesan A., Prabpal P. (2022). Numerical characterization of 1D-photonic crystal waveguide for female reproductive hormones sensing applications. Phys. B Condens. Matter.

[cit25] Pumera M. (2011). Graphene in biosensing. Mater. Today.

[cit26] Whitesides G. (2006). The origins and the future of microfluidics. Nature.

[cit27] Ouardi M. E., Meradi K. A., Tayeboun F. (2025). *et al.*, Development of a Novel SPR Biosensor for Early Pregnancy Detection. Sens. Imaging.

[cit28] Fan X., White I. M. (2011). Optofluidic microsystems for chemical and biological analysis. Nature Photonics.

[cit29] Masson J.-F. (2017). Surface plasmon resonance clinical biosensors for medical diagnostics. ACS Sens..

[cit30] Nguyen H. H., Park J., Kang S., Kim M. (2015). Surface plasmon resonance: a versatile technique for biosensor applications. Sensors.

[cit31] Sackmann E. K., Fulton A. L., Beebe D. J. (2014). The present and future role of microfluidics in biomedical research. Nature.

[cit32] Lončar M., Nedeljković D. (2019). Advances in nanophotonic biosensors using high-Q photonic crystal avities. Nat. Nanotechnol..

[cit33] Ahn S. R. (2020). *et al.*, Peptide hormone sensors using human hormone receptor-carrying nanovesicles and graphene FETs. Sci. Rep..

[cit34] Aly A. H., Awasthi S. K., Mohamed A. M., Matar Z. S., Mohaseb M. A., Al-Dossari M., Tammam M. T., Zaky Z. A., Amin A. F., Sabra W. (2021). Detection of Reproductive Hormones in Females by Using 1D Photonic Crystal-Based Simple Reconfigurable Biosensing Design. Crystals.

